# The effect of pre-operative exercise training on post-operative cognitive function: a systematic review

**DOI:** 10.1007/s41999-024-01028-4

**Published:** 2024-08-12

**Authors:** Hatice S. Ekici, Jemima Collins, Aysegul H. Kafadar, Mehmet C. Yildirim, Bethan E. Phillips, Adam L. Gordon

**Affiliations:** 1 Academic Unit of Injury, Recovery and Inflammation Sciences (IRIS), School of Medicine, University of Nottingham, Royal Derby Hospital, Uttoxeter Road, Derby, DE22 3NE UK; 2 Academic Unit of Mental Health and Clinical Neuroscience, School of Medicine, University of Nottingham, Nottingham, UK; 3 NIHR Nottingham Biomedical Research Centre, School of Medicine, University of Nottingham, Nottingham, UK; 4 NIHR Applied Research Collaboration East Midlands (ARC-EM), Nottingham, UK; 5 Department of Medicine of the Elderly, University Hospitals of Derby and Burton NHS Foundation Trust, Royal Derby Hospital, Derby, UK

**Keywords:** Cognition, Surgery, Exercise, Prehabilitation, Operative

## Abstract

**Background:**

With population aging and advances in surgical and anesthetic procedures, the incidence of surgery in patients over the age of 65 years is increasing. One post-operative complication often encountered by older surgical patients is post-operative cognitive dysfunction (POCD). Preoperative exercise training can improve the overall physiological resilience of older surgical patients, yet its impact on post-operative cognition is less well-established.

**Methods:**

Six databases (Medline (OVID); EMBASE (OVID); EMCARE (OVID); CINAHL (EBSCOHost), the Cochrane Library, and PubMed) were searched for studies reporting the effect of pre-operative physical training on post-operative cognition. The quality of evidence was assessed using the Mixed Methods Assessment Tool.

**Results:**

A total of 3983 studies were initially identified, three of which met the inclusion criteria for this review. Two studies were pilot randomized trials, and one was a prospective randomized trial. Two of the studies were high-quality. Each study used a different type of physical exercise and cognition assessment tool. Across the studies, post-operative cognition (*p* = 0.005) and attention (*p* = 0.04) were found to be better in the intervention groups compared to control, with one study reporting no difference between the groups.

**Conclusion:**

Preoperative physical training may improve post-operative cognitive function, although more research with a consistent endpoint is required. Future studies should focus on patients at high risk of POCD, such as older adults, and explore the impact of different exercise regimes, including frequency, intensity, time, and type.

**Supplementary Information:**

The online version contains supplementary material available at 10.1007/s41999-024-01028-4.

## Key summary points

**Aim:**

To investigate the effect of pre-operative exercise training on post-operative cognition.

**Findings:**

There are very few studies examining the effect of pre-operative exercise training on post-operative cognition. However, based on the results of previous studies, pre-operative exercise may increase post-operative cognition. More studies are needed to demonstrate the effect of pre-operative exercise training more clearly on post-operative cognition.

**Message:**

Preoperative exercise training is a practice that may be effective on post-operative cognition, and studies are needed to examine its effect on older adults.

**Supplementary Information:**

The online version contains supplementary material available at 10.1007/s41999-024-01028-4.

## Introduction

Globally, the number of people aged 60 years and older is increasing rapidly and is expected to reach 1.4 billion by 2030 and 2.1 billion by 2050 [1]. Advances in anesthetic and surgical procedures have made it easier to operate on older patients [2, 3], and the incidence of conditions requiring surgical intervention almost doubles with advancing age [4, 5]. Together, this means that older surgical populations are a growing clinical cohort. These populations pose unique challenges because of the prevalence of frailty and multimorbidity, which increases the likelihood of both pre- and peri-operative adverse events [3, 5].

One serious complication following surgery is post-operative cognitive dysfunction (POCD) [6, 7]. POCD is characterized by decreased attention, difficulties in processing information, disorientation, decreased perception, reduced consciousness, and memory disorders. Defined as a cognitive impairment that persists 30 days after surgery, based on a recognition that factors including emotional stress, pain, and medication can influence cognitive function earlier in the post-operative period [8], POCD can also cause both personality changes and mood disorders [9] in addition to cognitive impairment. In older adults, almost a quarter of patients aged over 65 years will experience a significant decrease in cognition after major surgery, with this becoming permanent in approximately half of these individuals [10]. Unsurprisingly, POCD has been shown to be associated with long-term declines in daily functioning [11, 12].

Away from the peri-operative setting, there is a wealth of literature demonstrating the positive impact of exercise training on multiple aspects of cognitive function [13–16] in both young [17] and older adults [18], with further evidence supporting the role of exercise training to reduce the risk of cognitive decline in later years [19, 20]. Within this, exercise is used as an umbrella term to describe various types of structured physical activity (e.g., aerobic, resistance, and balance-based exercise training such as tai chi), with different potential mechanisms underpinning cognitive improvement ascribed to each [16]. These mechanisms include improving brain plasticity [21, 22], increasing the level of growth factors involved in brain-derived neurotrophic factor (BDNF) and insulin-like growth factor 1 (IGF-1), regulating inflammatory cytokines [23, 24], increasing cerebral blood flow [25] and reducing oxidative stress [26]. However, despite this existent evidence base, to date, there has been no synthesis of the available evidence regarding the effect of pre-operative exercise training on post-operative cognition.

The EUGMS guidelines have recently been published, derived from a literature review, reported using the grading of recommendations, assessment, development, and evaluation (GRADE) framework, and the discussions of community experts to reach a consensus. The guideline strongly recommends the use of physical activity and exercise for the prevention and management of MCI and dementia, even if there is a weak evidence base for the positive cognitive effects of physical activity and exercise, as overall beneficial effects, both physical and psychological, have been demonstrated [27].

## Methods

### Search strategy and inclusion criteria

This systematic review was conducted according to the preferred reporting items for systematic reviews and meta-analysis (PRISMA) 2020 statement (Appendix 1). The review protocol was registered with PROSPERO (ID: CRD42022370648) before data extraction. A detailed summary of search methods and strategies is available in supplementary materials. Six electronic databases: Medline (OVID); EMBASE (OVID); EMCARE (OVID); CINAHL (EBSCOHost), Cochrane Library, and PubMed) were scanned in May 2024. These databases were searched with keywords including cognitive* impair*, cognitive function, surgery, operation, exercise, and exercise therapy. A full description of the search strategy for each database and the results obtained is available in Appendix 2.

Duplicates were identified and excluded using Rayyan software. Titles and abstracts of all articles were then screened for inclusion by two independent reviewers (H.S.E and A.H.K), with disagreements resolved by discussion with a third reviewer (A.L.G). Full-text review was completed by done by two independent reviewers (H.S.E and M.C.Y) with disagreements again resolved by discussed with a third author (A.L.G) until a consensus was reached.

All studies published in English exploring the effect of pre-operative physical exercise interventions on cognitive function in adults (over 18 years of age) undergoing emergency or elective surgery were included. Studies that only applied cognitive training were excluded. We included randomized controlled trials (RCTs), cluster RCTs, cohort studies, case–control studies, mixed-methods studies, and cross-over trials. We excluded case reports, editorials, commentaries, opinion papers, educational dissertations, and conference abstracts.

### Quality assessment (risk of bias)

Two reviewers (H.S.E and A.H.K) independently assessed the quality of included studies with disagreement resolved by discussion with a third reviewer (A.L.G) to reach a consensus.

The Mixed Method Assessment Tool (MMAT, version 2018) was used to assess quality considering randomization, baseline differences, completeness of data, blinding, and intervention adherence. The MMAT provides a quality score from 0 to 5, with a score of 4 or 5 considered high quality, and a score of < 3 low quality [28].

### Data extraction

Data extraction was performed by two independent reviewers (H.S.E and M.C.Y) using a preformed data extraction table (Appendix 3). Extracted data included first author, year of publication, country, study aims, study design, description of participants, interventions and comparator, type of surgery, POCD assessment method, time of test in relation to surgery, and outcomes. Data from the three included studies were synthesized and presented comparatively.

## Results

### Study selection

**Fig. 1:**
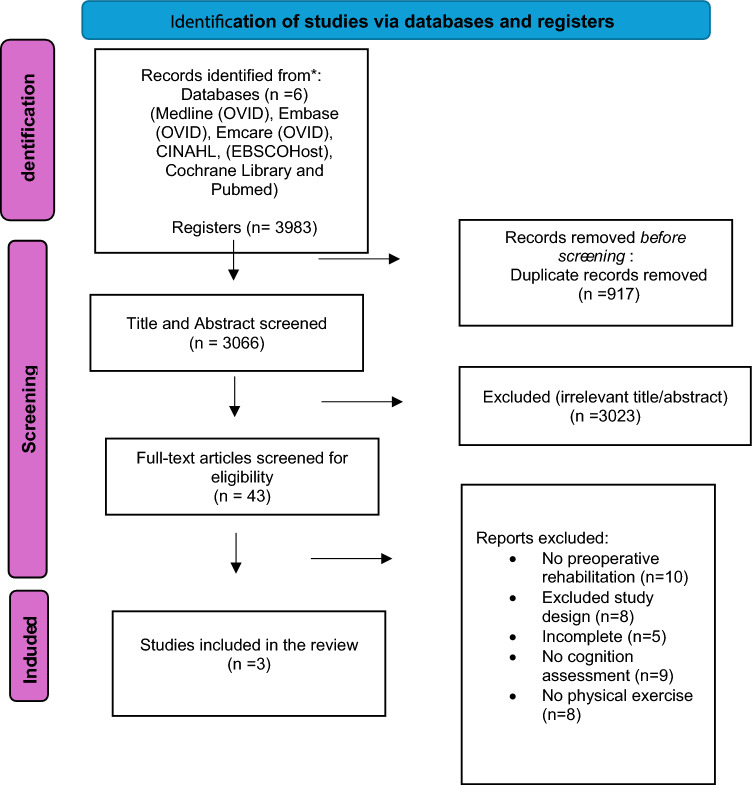
A PRISMA diagram. From: Page MJ, McKenzie JE, Bossuyt PM, Boutron I, Hoffmann TC, Mulrow CD, et al. The PRISMA 2020 statement: an updated guideline for reporting systematic reviews. BMJ 2021;372:n71. https://doi.org/10.1136/BMJ.n7

The initial search identified 3983 studies, which were reduced to 3066 following de-duplication. Title and abstract screening excluded a further 2303 articles, leaving 43 eligible for full-text screening. Nine of these were excluded because there was no assessment of cognition, eight because they adopted an excluded study design (case report, conference abstract, etc.), five because they reported an incomplete study, ten because the intervention took place post-operatively, and eight because no physical exercise training was included as part of the intervention. As a result, three studies were included in the final review [29–31]. This is summarized in a PRISMA diagram in Fig. 1.

### Study characteristics

Sample sizes of the included studies ranged from 25 to 103 participants. The mean age of the participants ranged from 44 to 73. Two of the three studies were conducted in the USA and one in Russia. Two of the studies were pilot randomized studies, and one was a prospective randomized study.

### Quality assessment

Two of the included studies were assessed as high quality with MMAT scores of 5 [31] and 4 [29]. The third study had an MMAT score of 3 [30], categorized as low quality. Issues related to lack of outcome assessor blinding [29, 30] and incomplete data [30] (Table 1).

**Table 1:** Quality assessment

	Is randomization appropriately performed?	Are the groups comparable at baseline?	Are there complete outcome data?	Are assessors blind to the intervention?	Did participants adhere to the intervention?	MMAT Score
Trubniko et al., 2021 [31]	Yes	Yes	Yes	Yes	Yes	5 (100%) High-quality
Kim et al., 2021 [29]	Yes	Yes	Yes	No	Yes	4 (80%) High-quality
Rengel et al., 2021 [30]	Yes	Yes	No	No	Yes	3 (60%) Low-quality

Quality assessment conducted using the mixed method assessment tool (MMAT)

### Study findings

The study by Kim et al., [29] explored the effect of pre-operative water-based (aquatic) exercise training on post-operative cognition in patients undergoing knee arthroplasty. The mean age of participants in this study was 67.1 (± 6.2) years, with 44% female. The exercise intervention lasted between 4 and 8 weeks, with assessments conducted after the intervention (pre-surgery) and between 4 and 6 weeks after surgery. The intervention was fully supervised and involved 3 sessions per week, with a stated aim of improving flexibility, strength and cardiovascular fitness. Those in the control group received only a brochure on peri-operative nutrition in addition to standard care. Cognitive function was evaluated using the montreal cognitive assessment (MoCA), a widely validated 30-point test that evaluates multiple domains of cognitive function including short-term memory recall tasks, visuospatial abilities, executive function, attention, concentration, working memory, language, and orientation [32]. MoCA score increased in the intervention group only at both the post-intervention (Mean [95% CI] Baseline: 25.5 ± 2.2, post-intervention: 27.2 ± 0.4, + 1.7, *p* = 0.0008) and post-operative (Mean [95% CI] Post-operative: 27.6 ± 0.4, + 2.1, *p* = 0.033) time points with an overall intervention effect of + 1.46 (95% CI 0.46, 2.45) *p* = 0.005.

The study by Trubnikova et al. [31] explored the effect of pre-operative aerobic exercise training on post-operative cognitive function in patients undergoing coronary artery bypass grafting (CABG). The mean age of participants was 58 (Q25; Q75 (52;65)) years and all participants were male. The intensity of the exercise intervention was individualized for each participant based on a pre-intervention cardiopulmonary exercise test but included supervised daily treadmill exercise for 40 min (including 5-min warm up and recovery phases) with a target rating of perceived exertion of 12–15 (“somewhat hard” to “hard”) based on a modified Borg scale [33]. The control group received no exercise intervention. The Mini-Mental State Examination (MMSE), Frontal Assessment Battery (FAB), and extended neuropsychological testing (the assessment of psychomotor and executive function, attention, and short-term memory from the neuropsychological complex software “Status PF” [34] were used to assess cognition 7 to 10 days after surgery. Incidence of cognitive decline was significantly lower in the exercise intervention group (58%) compared to control (79.5%) (OR: 2.74, 95% Cl 1.11–6.81, *p* = 0.029). In addition, the attention score of the exercise intervention group was significantly higher compared to control (*p* = 0.048). Further, the post-operative attention score of the exercise intervention group was higher than their pre-operative score (*p* = 0.04), while the control group had worse attention scores post-operatively (*p* = 0.03). There was however no difference between the two groups for psychomotor or executive function, with both groups showing significant improvements after surgery (*p* = 0.0008 and *p* = 0.0001, respectively). Finally, improvement in cognition was seen in the intervention group only (*p* = 0.06).

The final study by Rengel et al. [30] examined the effect of multimodal prehabilitation comprising cognitive and physical exercise on cognitive function in patients who underwent major non-cardiac surgery. The median age of participants was 62 years in the intervention group and 56 in the control group. Both groups in this study received generalized health information and also an electronic tablet on which they were asked to play a game daily. Referred to as the “active attention” group, the game played by the control group was a trivia-based electronic game, while that played by the intervention group was an adaptive and progressive game specifically designed to improve cognition. In addition, the intervention group received resistance bands and a prescribed exercise training plan using these bands. Both groups received weekly phone calls to provide support and answer questions. Cognitive function was evaluated using elements of the National Institute of Health (NIH) Cognitive Battery, one-month after surgery. Although patients in the intervention group tended toward improvement in cognitive scores (dimensional change card sort—a measure of executive function and shifting, flanker inhibitory control—a measure of executive function and attention, and pattern comparison processing—a measure of processing speed), these did not reach statistical significance.

A meta-analysis could not be performed because the three included studies had different interventions and different outcome measures.

## Discussion

This systematic review examined the effect of pre-operative exercise training on post-operative cognition. We found a paucity of literature in this area, with only three studies eligible for inclusion. Further, in each study, a different exercise training modality was used (aquatic exercise, aerobic exercise and combined [cognitive and aerobic] exercise) and different cognitive assessments employed. All three of the included studies found benefits for exercise prehabilitation on post-operative cognition.

Cognition is key for functional independence and effective communication [35, 36]. The cognitive decline seen with advancing age is associated with both structural and functional changes [37], and although this may be inevitable as part of normal aging [38], can be significantly accelerated by acute stressors including surgery [8] (i.e., POCD). Given the increased number of older patients presenting for surgery [4, 5] and the significant detrimental clinical and patient-centered impacts associated with POCD [6, 7], interventions to try and mitigate this should be afforded more attention.

One of the studies included in this review looked at the effect of pre-operative aquatic exercise on post-operative cognition and reported an improvement (via MoCA) using this intervention [29]. Further supporting the utility of aquatic exercise to improve cognition, a recent (2021) systematic review of 16 studies [18] reported aquatic exercise to have a positive effect on multiple neuropsychological factors in older adults, including cognition. Cohorts in this review include healthy community-dwelling older adults [39–43], care home residents with dementia [44, 45], older adults with mild-to-moderate Parkinson’s disease [46], older adults with a falls history [47], and older adults with a skeletal disorder (osteoporosis, osteopenia or osteoarthritis) [41–43, 48–50], highlighting the broad applicability of this type of intervention. Compared to land-based exercise training, water-based training may counter several barriers to exercise for older adults (e.g., fear of falling), with aquatic exercise shown to provide supported situations of instability and challenges to the center of gravity and postural control [51–53], each of which improve body balance reactions [51, 54]. A subsequent RCT of aquatic exercise training in older adults supported the utility of this intervention for improving not only cognition but also body composition and functional fitness. In addition, this study highlights how even within a single modality of exercise training (i.e., aquatic exercise), differences in activity profile can impact the physiological outcomes, with continuous and interval-based exercise most effective for improving functional fitness compared to a combination of these for body composition and cognition [55].

Beyond aquatic training, there is a wealth of literature demonstrating the positive effects of exercise training on cognition in both young [17] and older [18] adults, including systematic reviews [13, 15]. However, as highlighted by this review, there is little evidence to support the suggestion that pre-operative exercise training may improve post-operative cognition and/or the development of POCD. Although our included study of combined cognitive and resistance exercise training (30) was only able to demonstrate numerical trends toward improvements in cognition, a recent (2021) systematic review and meta-analysis [56] of 47 RCTs (41 for quantitative analysis) investigating the efficacy of combined cognitive and physical training interventions on cognitive, physical, psychosocial, and functional outcomes, concluded that combined training was superior to a single modality and that for cognition, simultaneous (compared to sequential or exergaming) training was the most effective. Of note, across the studies included in this systematic review, the physical components of these interventions were aerobic, resistance, or balance-based training, each of which is incorporated into current physical activity guidelines for older adults [57], based on their ability to elicit distinct physiological adaptation [58, 59]. As such it may be suggested that a regime incorporating each of these plus cognitive training, would best enhance the physiological resilience of older adults prior to surgery.

The aerobic exercise training-only study included in this review [31] did show a significant improvement in cognition in the exercise group, but the relevance of this study to POCD may be disputed given its assessment at only 7–10 days after surgery. In the initial days post-surgery, numerous factors, such as emotional burden, pain, and medication [8] with cognitive declines that resolve in this period have less impact on longer-term quality of life and return to normal activities.

One potential explanation for the disparity in significant effect between the aquatic [29] and aerobic [31] exercise studies included in this review, and the included study that utilized combined training [30] (beyond participant numbers), is exercise supervision. Although adherence was reported in all three studies, and each received a point for this criterion on the MMAT, the former two studies involved fully supervised exercise training, while the latter was home-based with weekly telephone “check-ins”. Supervised exercise has been shown superior to unsupervised exercise for adherence and compliance in many studies employing different types of exercise regimes across different cohorts [60–62]. However, evidence for this translating to better physiological adaptation is not unequivocal with, for example, supervision shown to have no impact on the physiological adaptation of older adults to 4-week high-intensity interval training [63].

A strength of this systematic review is that it provides a synthesis of all available evidence regarding the effect of pre-operative exercise interventions on POCD. We adhered to a pre-published protocol and systematically ascertained the risk of bias. We have reported our findings here in line with PRISMA guidance.

However, there are also some limitations. The first one of the included studies was low quality. Second, the other study included a specific population of male CABG patients, with implications for generalizability. It is possible that our stringent and systematic searching criteria, and the non-inclusion of gray literature, and non-English language literature, could have restricted the number of papers for inclusion.

In conclusion, this systematic review demonstrates that pre-operative exercise training has potential to improve post-operative cognitive dysfunction in adult patients undergoing elective or emergency surgery. With a limited number of studies exploring this impact, each using a different exercise training modality and cognitive assessment tool, further research should look to: (i) use a standardized assessment tool; (ii) assess the impact of interventions in older adults as those most at risk of POCD; and (iii) recruit larger cohorts to enable appropriate statistical analysis. Finally, given the increasing interest in surgical exercise prehabilitation by researchers, clinicians, and stakeholders, efforts should be made to explore the impact of exercise at this stage of the surgical journey on cognitive outcomes, especially in older adults, with a future aim of developing optimal exercise interventions that fit with existing clinical pathways.

## Supplementary Information

Below is the link to the electronic supplementary material.

Supplementary file1 (DOCX 496 KB)
