# Supplementary material for: The effect of pre-operative exercise training on post-operative cognitive function: a systematic review
Source: Eur Geriatr Med. 2024 Aug 12;15(5):1259–66. doi: 10.1007/s41999-024-01028-4 (PMC11614964; doi:10.1007/s41999-024-01028-4)
Supplement: Supplementary file 1 — Supplementary file1 (DOCX 496 KB) [file 41999_2024_1028_MOESM1_ESM.docx]

Appendix 1- PRISMA- Checklist


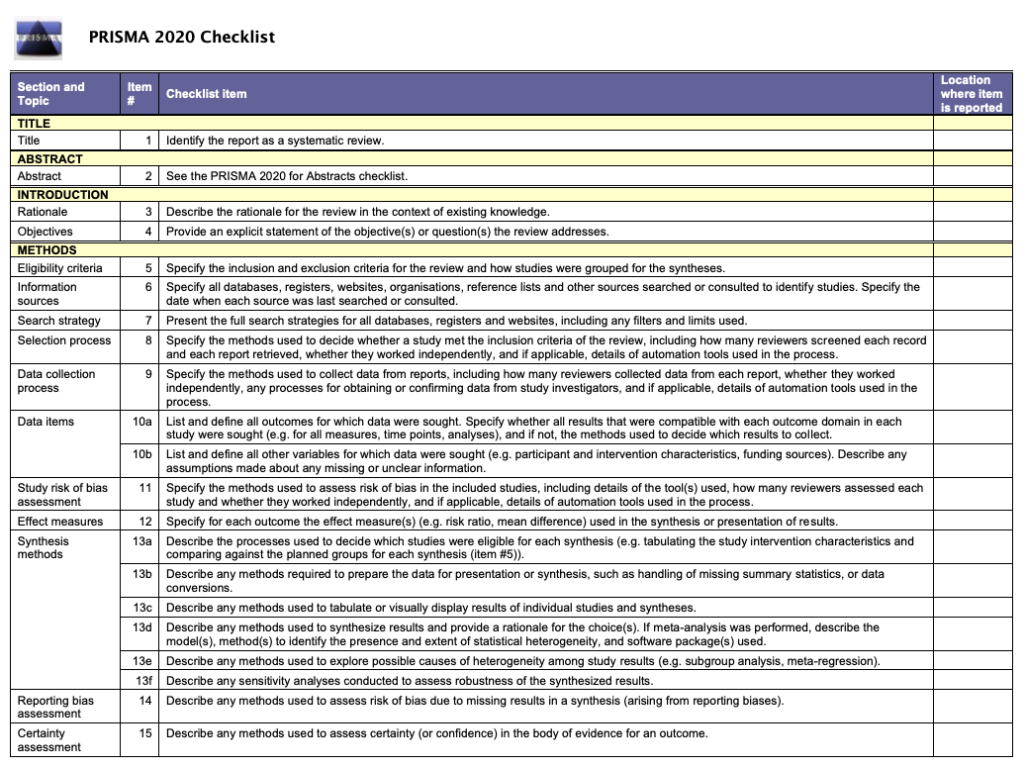


Appendix 2- For Methods section:

The searches were run in Medline (Ovid); EMBASE (OVID); EMCARE (OVID); CINAHL (EBSCOHost), the Cochrane Library ([www.thecochranelibrary.com](http://www.thecochranelibrary.com)) and PubMed on 16 May 2024. No date or language limits were applied. Full search strategies are in the appendices.

Ovid MEDLINE(R) ALL <1946 to May 24, 2024>

1 cognitive* impair*.mp. [mp=title, book title, abstract, original title, name of substance word, subject heading word, floating sub-heading word, keyword heading word, organism supplementary concept word, protocol supplementary concept word, rare disease supplementary concept word, unique identifier, synonyms] 86136

2 cognitive dysfunction/ 31842

3 cognitive dysfunction.mp. 46594

4 cognitive function.mp. 46145

5 1 or 2 or 3 or 4 137917

6 (surgery or surgical or operative).mp. 3469012

7 exp Surgical Procedures, Operative/ 3461499

8 6 or 7 4938395

9 5 and 8 9038

10 pocd.mp. 1109

11 Postoperative Cognitive Complications/ 390

12 10 or 11 1321

13 9 or 12 9370

14 exercise.mp. 408550

15 exp exercise/ 236284

16 exp exercise therapy/ 60810

17 14 or 15 or 16 473203

18 13 and 17 282

Embase <1974 to 2024 May 24>

1 cognitive* impair*.mp. [mp=title, abstract, heading word, drug trade name, original title, device manufacturer, drug manufacturer, device trade name, keyword heading word, floating subheading word, candidate term word] 137377

2 cognitive defect/ 197166

3 cognitive dysfunction.mp. 27522

4 cognitive function.mp. 69969

5 1 or 2 or 3 or 4 300565

6 (surgery or surgical or operative).mp. 4608952

7 exp surgery/ 5475603

8 6 or 7 6514210

9 5 and 8 31139

10 pocd.mp. 1492

11 postoperative cognitive dysfunction/ 1706

12 10 or 11 2346

13 9 or 12 31509

14 exercise.mp. 573256

15 exp exercise/ 401247

16 exp kinesiotherapy/ 92926

17 14 or 15 or 16 622875

18 13 and 17 1359

Ovid Emcare <1995 to 2024 Week 37>

1 cognitive* impair*.mp. [mp=title, abstract, heading word, drug trade name, original title, device manufacturer, drug manufacturer, device trade name, keyword heading word] 46895

2 cognitive defect/ 57698

3 cognitive dysfunction.mp. 6955

4 cognitive function.mp. 23737

5 1 or 2 or 3 or 4 96985

6 (surgery or surgical or operative).mp. 899063

7 exp surgery/ 1164372

8 6 or 7 1387194

9 5 and 8 8648

10 pocd.mp. 495

11 postoperative cognitive dysfunction/ 603

12 10 or 11 857

13 9 or 12 8771

14 exercise.mp. 217393

15 exp exercise/ 171359

16 exp kinesiotherapy/ 39618

17 14 or 15 or 16 238795

18 13 and 17 358

EBSCOHost CINAHL

| S1 | TI (cognitive* n1 impair*) OR AB (cognitive* n1 impair*) | 33,039 |
| --- | --- | --- |
| S2 | TI ((cognitive n1 dysfunction) OR (cognitive n1 function)) OR AB ((cognitive n1 dysfunction) OR (cognitive n1 function)) | 22,966 |
| S3 | (MH "Cognition Disorders") | 33,426 |
| S4 | S1 OR S2 OR S3 | 67,316 |
| S5 | TI (surgery or surgical or operative) OR AB (surgery or surgical or operative) | 459,454 |
| S6 | (MH "Surgery, Operative+") | 749,939 |
| S7 | S5 OR S6 | 961,870 |
| S8 | S4 AND S7 | 3,653 |
| S9 | TI (pocd) OR AB (pocd) | 264 |
| S10 | S8 OR S9 | 3,692 |
| S11 | TI (exercise) OR AB (exercise) | 134,092 |
| S12 | (MH "Exercise+") | 128,584 |
| S13 | (MH "Therapeutic Exercise+") | 61,851 |
| S14 | S11 OR S12 OR S13 | 223,271 |
| S15 | S10 AND S14 | 136 |

Cochrane Library

ID Search Hits

#1 (cognitive* near impair*):ti,ab,kw 13338

#2 (cognitive near dysfunction):ti,ab,kw 4724

#3 (cognitive near function):ti,ab,kw 13652

#4 MeSH descriptor: [Cognitive Dysfunction] this term only 2274

#5 #1 or #2 or #3 or #4 25390

#6 (surgery or surgical or operative):ti,ab,kw 286389

#7 MeSH descriptor: [Surgical Procedures, Operative] explode all trees 129624

#8 #6 or #7 332440

#9 #5 and #8 2403

#10 pocd:ti,ab,kw 415

#11 MeSH descriptor: [Postoperative Cognitive Complications] explode all trees 47

#12 #9 or #10 or #11 2489

#13 exercise:ti,ab,kw 113499

#14 MeSH descriptor: [Exercise] explode all trees 28693

#15 MeSH descriptor: [Exercise Therapy] explode all trees 16366

#16 #13 OR #14 OR #15 117521

#17 #12 AND #16 169

PubMed

((((cognitive impairment) OR (cognitive function) OR (cognitive dysfunction) OR ("cognitive dysfunction"[MeSH Terms])) AND (surgical OR surgery OR operative OR (surgical procedures, operative[MeSH Terms]))) OR POCD OR ("postoperative cognitive complications"[MeSH Terms])) AND (exercise OR ("exercise"[MeSH Terms]) OR ("exercise therapy"[MeSH Terms]))

Appendix 3- Charted data of findings

| **First author, year** | **Country** | **Study aim (synopsis)** | **Study design** | **Participants** | **Interventions**  **(Exercise type/duration)**  **Comparator** | **Type of surgery** | **Cognitive assessment method** | **Post surgery test time** | **Outcomes** |
| --- | --- | --- | --- | --- | --- | --- | --- | --- | --- |
| Kim et al., 2021(28) | USA | To examine the effect of a preoperative aquatic exercise intervention on postoperative outcomes in older adults undergoing total knee arthroplasty. | Pilot randomized clinical trial. | N= 43  (19F, 24M)  >50 years old  Mean age: 67.2±6.1 years  Scheduled for elective total knee arthroplasty  Below 50^th^ percentile for mobility | **Intervention group (N=20)**  Standard care plus 4 to 8 weeks of aquatic exercise intervention:  three times each week  60 min per session (10 min warm-up, 20 min flexibility & strength exercises, 20 min low intensity aerobic exercise, and 10 min cool down).  **Control group (N=23)**  Standard care plus a perioperative nutrition brochure. | Total knee arthroplasty | Montreal Cognitive Assessment (MoCA) | 4-6 weeks after surgery | Aquatic exercise was also associated with improved cognition, measured by MoCA.  The MoCA score of the intervention group improved by 1.7 and 2.1 post-intervention and postoperatively, respectively.  There was no change in MoCA score in the control group. |
| Rengel et al., 2021 (29) | USA | To investigate the postoperative outcomes of home-based combined physical and cognitive prehabilitation on non-cardiac surgery patients. | An open-label, randomised, controlled pilot feasibility trial. | N=25  ≥18 years of age  Scheduled for major non-cardiac  surgery requiring ≥3 days of hospitalization  Intervention group (N=17) Mean age: 62 years (IQR: 50-69)  Control group (N=8)  Mean age: 56 years (IQR: 44-66.5) | **Intervention group (N=17)**  10 to 21 days of computerised  cognitive training game from PositScience on a provided tablet, with participants requested to play each day.  General health information.  Resistance exercise training-based plan and resistance bands provided, with daily intervention scheduled.  Weekly phone calls to monitor intervention engagement.  **Control Group (N=8)**  10 to 21 days of computerised  trivia-based training game on a provided tablet, with participants requested to play each day.  General health information.  Weekly phone calls to monitor intervention engagement. | Non-cardiac surgery | Aspects of the National Institute of Health (NIH)  Cognitive Battery:  Dimensional Change Card Sort  Pattern Comparison Processing  Flanker Inhibitory Control | 1 month after surgery | 88% of participants in the intervention group and 87% in the control group performed at least one of the assigned activities each day during the preoperative period.  Patients in the intervention group tended towards an improvement in cognitive scores.  No statistically significant difference in cognition between the intervention and control groups. |

| Trubnikova et al., 2021 (30) | Russia | To determine how a physical prehabilitation program affected cognitive function, electroencephalogram, and neurovascular unit markers in patients undergoing coronary artery bypass grafting. | Prospective  randomized study. | N= 103  ≥45 years old without depression or dementia  Intervention group (N= 50)  Mean age:  59 years  (range: 52-65)  Control group (N=53)  Mean age:  58 years (range: 53-63) | **Intervention Group:**  Aerobic  exercise training for 5 to 7 days:   - Daily   40 min per session (5 min warm-up, 30 min exercise with intensity based on pre-intervention cardiopulmonary exercise test and aiming to achieve a rating of perceived exertion between 12-15 (Borg scale), 5 min cool down).  **Control group:**  Standard preoperative preparation. | Coronary artery bypass grafting | Mini Mental State Examination (MMSE)  Frontal Assessment Battery (FAB)  Extended neuropsychological testing | 7–10 days after surgery | Postoperative cognitive dysfunction (POCD) occurred in 58% of the intervention group and 79.5% of the control group.  Attention scores were higher in the intervention group than the control group.  The intervention group had an improved attention score postoperatively, while the control group had a worse attention score.  No significant difference was found between the groups in psychomotor or executive function.  The integral indicator of cognitive status improved only in the intervention group.  Incidence of POCD decreased in the intervention group only. |
| --- | --- | --- | --- | --- | --- | --- | --- | --- | --- |

**Revision Sheet**

For authors to describe revisions made in response to peer review comments.

**Manuscript Title: The effect of preoperative exercise training on postoperative cognitive function: a systematic review**

| **Reviewer 1 comments:** | | |
| --- | --- | --- |
| Comment raised | Response by author | Location of revisions |
| “I suggest adding the means to the results section of the summary” | I added the overall intervention effect score of a study.  However, in Trubnikova's study, the results were shown in figures, and I could not add the mean value. | Abstract |
| “On page 12, in the Results section, it is advisable to add the means of the comparisons made for the Student's t-test for related samples and the Student's t-test for independent samples, to see the magnitude of the difference” | Thank you, I added Mean values. | Results  Page 12 |
| “Additionally, on page 12, in rows 45 to 52, it is advisable to mention the means of the scores and express them in p-values with their confidence intervals, as the lack of differences may be due to the small sample sizes. “ | In the article, p values are shown in the figure. Mean values are not displayed. | Results  Page 12 |

| **Reviewer 2 comments:** | | |
| --- | --- | --- |
| Comment raised | Response by author | Location of revisions |
| I noticed that the EUGMS guideline named "Physical activity and exercise for the prevention and management of mild cognitive impairment and dementia: a collaborative international guideline" was not included in the justification or introduction of the review. | We have added this. | Introduction  Last Paragraph |

| **Editor comments:** | | |
| --- | --- | --- |
| Comment raised | Response by author | Location of revisions |
| “The abstract of this article was presented at the 19th EuGMS congress and published in the abstract book. This has to be mentioned (perhaps in the acknowledgment section)” | Thank you, we have mentioned it accordingly. | Acknowledgments |
| “The last reference is from 2022. Please check the last two years for any article worth mentioning in the introduction/ discussion” | I have added the EUGMS guideline (2023). | Introduction  Last Paragraph |
| “I am not sure that the first paragraph of the introduction is needed, this is a geriatric journal, readers know about population aging and increasing amounts of patients .” | We reviewed this paragraph and felt that it provided necessary context for readers who may be new to the field.  Many readers of this article may not be subscribers to European Geriatric Medicine, and we felt it was important to keep the opening paragraph for this group of readers. | Introduction  First Paragraph |
| **“**Please carefully review the numbers of the references. Those cited in table 1 do not seem to correspond to the articles in the reference list. “ | Thank you, we have done this | Results,  Quality Assessment |
| “Do not use subheadings in the discussion section” | Thank you, we have done this | Discussion |
| “Many references are wrongly cited (including, but not only, journal abbreviations)” | Thank you, we have done this | References |

**Important Note:** The systematic review was updated in May 2024. There are no new studies that meet the inclusion criteria.
